# Neighborhood Differences in Omnipresent Policing and Sleep Health in New York City: Protocol for a Multimethod Quantitative Study

**DOI:** 10.2196/82605

**Published:** 2025-12-30

**Authors:** Martin J Downing Jr, Mia Budescu, Nicole M Holbrook

**Affiliations:** 1 Lehman College, City University of New York Bronx, NY United States; 2 Fordham University Bronx, NY United States

**Keywords:** protocol, sleep health, omnipresent policing, surveillance, neighborhood segregation

## Abstract

**Background:**

Poor or insufficient sleep is associated with numerous adverse, potentially serious physical and mental health outcomes. Equally concerning are the substantial racial, gender, and socioeconomic disparities, with minorities and those experiencing poverty disproportionately affected by poor sleep quality and sleep disorders. Both theory and research suggest that sleep health is negatively impacted by concentrated poverty at the neighborhood level due to the deterioration of the built and social environments, thereby creating conditions that disrupt sleep.

**Objective:**

This research considers an under-studied factor related to these conditions and sleep health—policing and police surveillance. Specifically, the study compares 4 neighborhoods within New York City at different levels of residential segregation.

**Methods:**

The study design consists of a baseline survey, with 40 residents recruited in each neighborhood, and a 1-week diary phase with a subsample of residents. Neighborhood conditions are also assessed in each of the neighborhoods using a neighborhood audit tool.

**Results:**

The study received funding in July 2024. Data collection commenced in September 2024. As of August 2025, we have enrolled more than 100 participants in the baseline survey. Planned analyses will begin once data collection has concluded.

**Conclusions:**

This information will help establish the extent to which surveillance and policing differentially impact the lives of New Yorkers as a function of where they live. Specifically, the results should be relevant and important for understanding the impact of novel policing strategies on underprivileged neighborhoods. This exploratory research will be useful for identifying populations and residential settings that may be most at risk for poor sleep health.

**International Registered Report Identifier (IRRID):**

DERR1-10.2196/82605

## Introduction

### Background and Rationale

Poor sleep health is considered a public health threat in the United States [1]. Among US adults, the prevalence of self-reported sleeping problems is 56%, whereas that of insomnia is estimated to be 27% [1,2]. Moreover, the prevalence of moderate to extreme insomnia symptoms suggests that this sleep disorder is underdiagnosed [3]. A 2020 poll [4] found that approximately half of Americans felt sleepy anywhere from 3 to 7 days a week, and for many, this sleepiness impeded their daily activities. Poor or insufficient sleep is associated with numerous adverse, potentially serious, physical and mental health outcomes [5-7]. Equally concerning are the substantial racial, gender, and socioeconomic disparities in sleep health [8-11]. Theory and research suggest that sleep disparities are partially driven by residential segregation or spatial and neighborhood separation along racial, ethnic, and socioeconomic lines [12]. This segregation shapes the physical (eg, crowding and walkability), social (eg, disorder and safety), and ambient (eg, light and noise pollution) environments, thereby contributing to poor sleep [13-15]. This study considers an under-studied factor related to sleep health, policing—specifically, police surveillance.

In the United States, communities of color are burdened by overpolicing and police brutality [16,17], which contribute to stress [18], adverse mental health outcomes [19,20], and poor sleep [21]. In recent decades, police departments in the United States have shifted toward omnipresent policing, which includes traditional ground presence as well as covert surveillance and deterrence strategies [22]. Examples include the placement of floodlights and surveillance towers in high-crime areas, increased undercover police presence, reliance on closed-circuit television, the use of drone footage and facial recognition software, and the interception of cellphone data. The New York City (NYC) Police Department recently vowed to expand its use of police surveillance [23]. Data indicate that surveillance is more common in communities of color [22], and footage obtained from thousands of drivers demonstrates that the NYC Police Department deployed more police to patrol low-income areas with higher Black and Latino populations [24]. It appears that omnipresent policing varies as a function of residential segregation, much like traditional policing, but little research has systematically examined cross-neighborhood differences or the impact of these strategies on sleep health.

These strategies are thought to influence sleep through physical, social, and ambient environments. Specifically, increased police presence contributes to noise and light pollution (eg, sirens and police lighting). For example, floodlights placed in areas identified as high crime [25] may contribute to light pollution and, in turn, worsen sleep health outcomes [26,27]. The principal investigators in this study previously conducted a small-scale survey on omnipresent policing in the Bronx borough of NYC and found that police surveillance evokes complex feelings of both safety and unease, which are emotional responses known to impact sleep quality [28-30]. Our pilot data similarly found that residents of the Bronx who are more worried about neighborhood crime safety, especially those identifying as Hispanic or Latino, reported more insomnia symptoms [31]. In contrast, feelings of unease related to being surveyed may lead to stress and negative emotions, thereby having a negative impact on sleep [12]. The complex interplay of these factors may also influence residents’ propensity to spend time outdoors and engage in social and physical activities, which have a known impact on sleep health [14]. For example, O’Connor and Jahan [32] found that Muslims who were targets of post-9/11 surveillance reacted more with anxiety than anger and modified their behaviors to avoid situations that might lead to further monitoring.

Previous theoretical models and research suggest that the neighborhood context is associated with sleep health [12,14]. However, these studies have not accounted for policing and specifically, surveillance, as a symptom of segregation. Studies on policing have largely relied on national samples, making it difficult to control for city- or county-level differences in crime levels and law enforcement policies. Finally, previous models [12] have relied on socioeconomic features to measure neighborhood disadvantage, not accounting for the confluence of race and socioeconomic status (SES) in shaping urban neighborhoods [33,34]. This study aims to address these gaps by surveying residents of, and comparing, 4 neighborhoods within NYC, at low, medium, and high levels of residential segregation, as measured by the Index of Concentration at the Extremes (ICE). The ICE race-income subset is a segregation measure that simultaneously accounts for race and SES [35]. The study also includes a 7-day diary period in which a subset of participants, equally represented across the neighborhoods, are asked to report on police interactions (both covert and overt) throughout the day as well as their sleep quality the same night.

Diary data are important since there is very little data on how often police interactions occur daily and their immediate impact on sleep and psychological health. Additionally, this design will help disentangle the extent to which residents of particular neighborhoods share similarities among themselves while being distinct from residents of neighborhoods with different levels of segregation, in terms of their police experiences and sleep health. This information can establish the extent to which surveillance and policing differentially impact the lives of New Yorkers as a function of where they live. Additionally, the study considers whether individual-level variables, such as household SES, psychological, and physical characteristics, exacerbate or mitigate neighborhood-level forces, with the hopes of informing future interventions. Importantly, this study also serves as a methodological pilot to determine the feasibility of eventually including a wider breadth of NYC neighborhoods for a more holistic understanding of sleep health and policing in the city.

### Conceptual Framework

**Figure 1:**
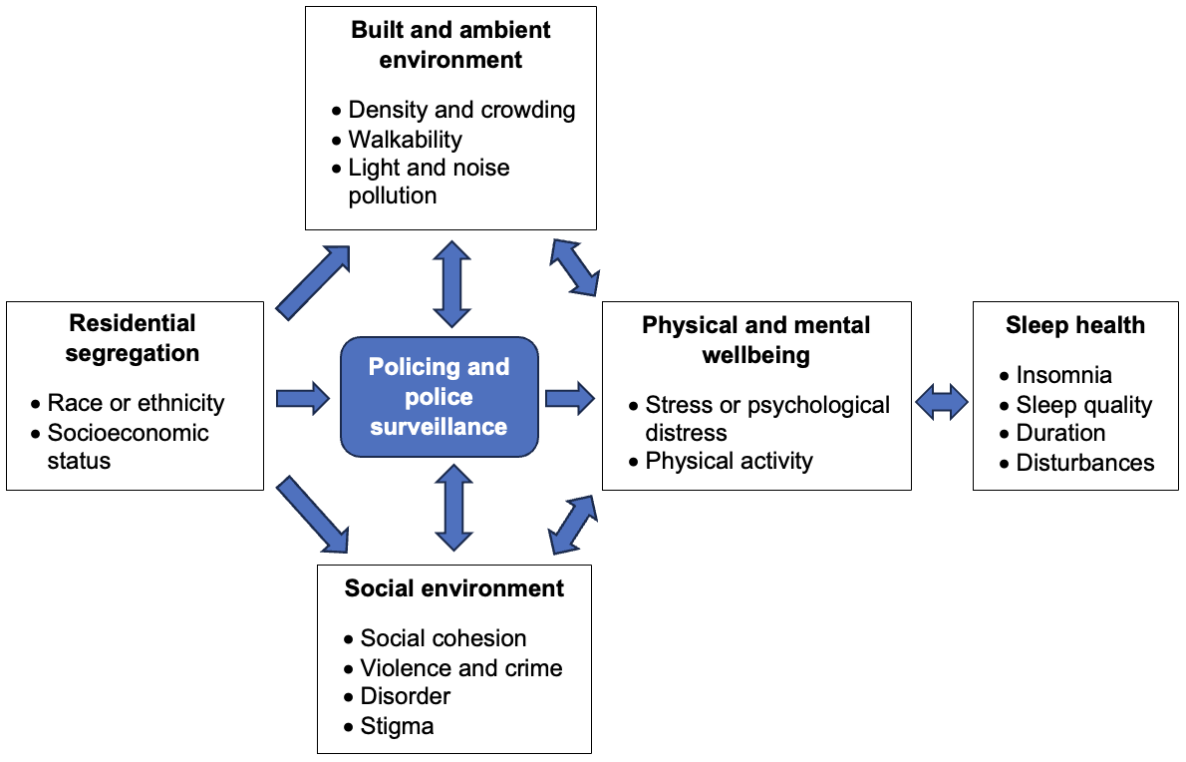
Modified conceptual framework.

This formative study applies a modified conceptual framework [12] positing that neighborhood segregation is associated with sleep health through the built and physical, social, and ambient environments, with a unique emphasis on policing (Figure 1). We are comparing 4 neighborhoods within a single city (ie, NYC), allowing us to control for variations in city-level policies that dictate law enforcement decisions. Not only are between-neighborhood differences relevant to understanding sleep health, but research is equally needed to address within-neighborhood variations (eg, why some residents may be more or less affected by the symptoms of neighborhood segregation). To test this framework, we are using a multilevel approach to examine between- and within-neighborhood differences in policing in relation to sleep health data.

### Study Goals

#### Goal 1

This study aims to investigate between-neighborhood differences in self-reported sleep health, including perceived sleep quality and insomnia, as a function of policing, both overt and covert. The following hypotheses are proposed:

1. The neighborhoods with higher levels of residential segregation (across racial-SES lines) will also have poorer overall sleep health and higher levels of policing compared with their less segregated counterparts.
2. Consistent with Figure 1, the association between policing and sleep health will be mediated by related neighborhood characteristics, including features of the built, social, and ambient environments.

#### Goal 2

This study aims to investigate individual (person-level) differences in sleep health, as a function of neighborhood and individual-level factors, to test two hypotheses:

1. Using baseline, cross-sectional data, it is hypothesized that individuals with more negative interactions and sentiments related to omnipresent policing will report lower levels of self-reported sleep health.
2. It is expected that individual-level factors (eg, household income, gender, race, ethnicity, education, and mental and physical health) will moderate the association identified in the previous hypothesis. Using intensive longitudinal data (the 1-week diary study), it is predicted that negative policing interactions on any given day will be associated with poorer self-reported sleep health on the same night.

## Methods

### Study Design

This study consists of a cross-sectional, baseline survey (phase 1; n=160) and a daily diary (phase 2; n=72). Given the preliminary nature of this research, eligible participants must be aged at least 18 years, fluent in English, and own a smartphone. Although these exclusions limit the generalizability of the results to the larger NYC population, they are deemed necessary given the budget and time constraints of this study.

### Neighborhood Tabulation Areas

We generated a list of all 197 neighborhood tabulation areas (NTAs) in NYC. These catchment areas are approximations of NYC neighborhoods created for the purpose of reporting decennial census and American community survey data. Then, we calculated measures of income and racial segregation for each NTA using the ICE for race-income [35]. ICE captures the degree to which residents are concentrated into areas with extreme deprivation or privilege and has a possible range of values from –1 (100% of the population consists of the deprived group) to +1 (100% of the population consists of the privileged group) [35]. ICE (income) is computed as follows*: ICE_income_* = (*A_i_ –P_i_*)/*T_i_*. *A_i_* is the number of persons in the top 80th percentile for income or higher (>$150K), *P_i_* is the number of people in the bottom 20th percentile (<$25,000), and is the total NTA population*.* ICE (race) is computed by subtracting the number of non-White residents from the number of White residents and then dividing by the population size [35]. We selected a random sample of 4 NTAs: one in the lowest quartile (Highbridge), 2 in the middle quartiles (Morningside Heights and Bensonhurst), and one in the highest quartile (Midtown South, Flatiron, and Union Square) on both ICEs. These NTAs represent 3 of the 5 NYC boroughs.

### Participant Flow: Recruitment, Baseline Survey (Phase 1), and Daily Diary (Phase 2)

We are conducting a street-intercept baseline survey (phase 1) with 40 residents from each of the 4 randomly selected NTAs (total baseline survey sample size=160). A street-intercept approach generates robust response rates when capturing specific health behaviors [36,37]. Moreover, this methodology is effective in obtaining crucial public health information about hard-to-reach communities. The survey, hosted on Qualtrics, is completed on the spot on a tablet and takes approximately 15 minutes to complete. The principal investigators for this project previously conducted street-intercept surveys with approximately 200 Bronx residents during 1- to 2-hour recruitment shifts across 23 nonconsecutive days over 1 year [31].

Recruitment takes place outdoors at high-volume locations, such as public parks, sidewalk intersections, and parking lots. Interested individuals are briefly screened, including verifying that they live in the target NTA. This is done by having participants identify where they live in the target NTA on a digital map [38] and tracking each participant’s census block. Eligible individuals provide informed consent before completing the survey using a study tablet. We will end recruitment in each NTA once the target goal of 40 participants, including 18 randomly selected for the daily diary, is reached.

Street-intercept recruitment efforts can be affected by weather conditions. On days with cooler temperatures, study team members screen potential participants during street-intercept recruitment and collect an email address for eligible persons willing to complete the survey. The lead investigators follow up with a same-day email that includes a link to the baseline survey, the recipient’s census block (as determined during screening) to enter in the survey, and information about how the US $15 compensation is provided.

### Daily Diary Procedures (Phase 2)

Following completion of the baseline survey, a subsample of 72 participants (n*=*18 from each NTA) is randomly selected for a 1-week daily diary consisting of 2 short daily surveys (morning and evening) hosted on Qualtrics. Enrolled participants begin their diary period approximately 24 to 96 hours after the baseline survey. The daily diary consists of 14 short (about 5 min) surveys (2 per d, for 7 d). Nightly surveys are completed between 7 PM and 3 AM, and interactions with the police and other daily stressors are assessed. Morning surveys are completed at any time between 5 AM and noon and assess sleep quality the night before. Every evening at 7 PM and morning at 5 AM, participants receive a text message to their personal smartphone with a link to that survey, hosted on an online survey platform (Qualtrics). Eligible participants must have their own smartphone that can connect to the internet. Although this may limit generalizability, recent Pew Research Center data suggest that 91% of US adults have a smartphone [39].

### Ethical Considerations

The study received approval (2024-0453-Lehman) from the institutional review board affiliated with the City University of New York. Individuals who screen eligible for the baseline survey provide informed consent on a study tablet before completing the survey. Following completion of the baseline survey, anyone who is randomly selected for the 1-week daily diary and agrees to participate is asked to provide informed consent on a study tablet. The internet-based consent forms for the baseline survey and daily diary state the voluntary nature of this study, indicate that the risks of participating are minimal, and specify that the participant does not have to answer any question that they do not want to answer. Diary participants provide their mobile phone number and a name to receive daily text messages with links to the surveys. For the purpose of participant validation, they are asked to re-enter this information on each daily survey. To maintain privacy and confidentiality, any participant contact information will be separated from the survey files and deleted at the conclusion of the study. Participants receive US $15 as compensation for their time for completing the baseline survey. For the diary phase, participants receive US $2.50 for completing their daily surveys (US $1.25 each) plus a US $7.50 bonus for completing the 7 days for a potential total of US $25. Completed surveys are automatically recorded and stored securely by the survey host.

### Study Measures

For the baseline survey, we are using validated instruments to measure sleep health (primary outcome), stress, neighborhood walkability, and social cohesion (Table 1). As the primary outcome for this study, sleep health will be captured using a composite score based on 4 indicators from the baseline survey, as shown in Table 1. The baseline survey also includes measures of noise, neighborhood reputation, perceived police surveillance, and over- and underpolicing, as well as perceptions of violent and property crime. We added a measure of police surveillance strategies (eg, awareness of and emotional reactions to) developed for a previous study of Bronx residents. The daily diary surveys include measures of sleep health, psychological distress, discrimination, stressful events, somatic symptoms, and police interactions (Table 1). We also added several items from the National Sleep Foundation diary (eg, sleep and wake times, nap taking, and exercise) [40].

**Table 1:** Study measures using validated instruments and measures adapted for this study.

Domain and measure			Study phase		
			Baseline		Diary
**Sleep** **health**					
	Subjective sleep quality [41]	✓		✓	
	Insomnia severity [42]	✓			
	Sleep hygiene [43]			✓	
	Daytime dysfunction [41]	✓			
	Use of pharmacological sleep aids [41]	✓			
**Stress and** **physical activity**					
	Psychological distress [44]	✓		✓	
	Perceived stress [45]	✓			
	Everyday discrimination [46]	✓		✓	
	Daily stressful events [47]			✓	
	Daily somatic symptoms^a^ [48]			✓	
	Recreational activities [49]	✓			
**Neighborhood** **environment** **and** **policing**					
	Walkability, lighting, and crime safety [50]	✓			
	Noise	✓			
	Social cohesion [51]	✓			
	Neighborhood reputation or spatial stigma [52]	✓			
	Perceived police surveillance (adapted) [53]	✓			
	Awareness of, and emotional reactions to police surveillance (Budescu M, unpublished data, June 2025)	✓			
	Over- and underpolicing [54]	✓			
	Perceptions of violent and property crime [55]	✓			
	Police interactions [56]			✓	
**Covariates**					
	Crime victimization	✓			
	Substance use [57,58]	✓			
	Sex at birth	✓			
	Gender identity	✓			
	Sexual orientation	✓			
	Ethnicity	✓			
	Race	✓			
	Employment status	✓			
	Household income	✓			
	Education	✓			
	Cohabiting with a romantic partner	✓			
	Children under the age of 18 or dependents	✓			
	Born in the United States (or Puerto Rico, Guam, United States Virgin Islands, and other US territories)	✓			

^a^We assessed the presence of several daily somatic symptoms, including abdominal pain or gastrointestinal issues, tingling or numbness, dizziness or fainting, and headache [48]. Our list also included changes in appetite and whole-body aches.

### Neighborhood Audit

Studies of neighborhood environment quality (eg, physical incivilities, defensible space, natural elements, and surveillance) have relied, at least in part, on the use of a neighborhood audit tool. Examples include the Residential Environment Assessment Tool 2.0 [59], the Built Environment Assessment Tool [60], the Microscale Audit of Pedestrian Streetscapes [61], and the Block Environmental Inventory [62]. These tools measure the physical environment of urban residential areas via on-the-ground observations by trained raters who look for and document evidence of block-level cues, social (eg, presence of people outside) and physical (eg, presence of abandoned cars, sidewalks, and crime watch signs) environment, as well as information about residential and nonresidential properties. The study investigators, along with trained research assistants, make assessments of neighborhood conditions using a modified version of the Revised Block Environmental Inventory [63].

We modified the Revised Block Environmental Inventory in several ways: fewer person entries (maximum 5), revised age categories for person entries (ie, child, adolescent, adult, or older adult [>60 years]), no sex categories for person entries, fewer nonresidential land use entries (maximum 3), and fewer residential property entries (maximum 3). We added several block items [64,65], such as grooves or bumps in the curb, path obstructions (eg, trees and poles), garbage cans, benches, water fountains, bicycle parking and lanes, public spaces (eg, playground or garden), pedestrian crossing signs or activated signals, transit stops (eg, bus stop), outdoor public dining areas, as well as the number of sidewalk cracks (defined as substantially hazardous for walking), speed bumps in the street, accessibility options (eg, ramp), and pits containing trees. For the modified version, raters assessed the block for aesthetics (ie, attractive for walking and cycling) and feelings of safety (for walking and cycling) [64]. For nonresidential land use properties, we assessed evidence of vandalism in addition to the original graffiti item. Finally, we added several police surveillance items to the audit, including floodlights, towers (to monitor and record ground movements), vans, cameras, ShotSpotters (ie, sound sensors), and in-person police presence.

First, we identified all census blocks within each of the 4 NTAs [66] and generated a random set of 2 census block codes for each (n=8 blocks). We included one additional census block in each NTA (a total of 3 blocks per NTA) that was not randomly selected but rather based on the study team’s familiarity with the neighborhood as part of our street-intercept recruitment (described earlier). The study team conducted several training sessions, which led to many of the modifications noted above. Using best practices [60,61], we are conducting observations (during team shifts) of the social (eg, presence of people outside) and physical (eg, presence of abandoned cars, sidewalks, and crime watch signs) environment, including gathering information about residential and nonresidential properties (eg, broken windows, litter, gardens), as well as looking out for known signs of police surveillance (eg, sound sensors, floodlights, towers, police presence). Auditors also measure census block noise levels using a sound level meter app installed on study tablets. This is done 3 times during each block audit. The sound level meter app was developed by the National Institute for Occupational Safety and Health to measure noise in the workplace and can be used anywhere for accurate noise measurement [67,68].

### Data Analysis

For goal 1, we will test 3 serial mediation models to estimate the direct and indirect pathways between neighborhood segregation and sleep health. Each model will consist of 2 serial mediators between these 2 variables: (1) policing and (2) a variable capturing either the built and physical, social, or ambient environment. Sleep health will be the primary outcome for these analyses and will be captured using a composite score based on 4 indicators from the baseline survey as shown in Table 1, which includes subjective sleep quality, insomnia severity, daytime dysfunction, and use of sleeping medication. A Monte Carlo power analysis for indirect effects for a model with 2 serial mediators [69] should have adequate power (0.78-0.82) for a sample size of 160 participants, assuming a moderate correlation between the predictor and mediators.

For goal 2, the diary data will be analyzed using multilevel modeling in which the 14 observations (7 nights and mornings) are nested within the individual respondents [70]. This approach will allow us to test within- and between-subject effects simultaneously. Within-subject analyses will address whether participants report worse or better sleep health than on previous days, depending on experiences they had that day, as well as potential lagged effects. The between-subject analyses will address whether certain baseline characteristics predict individual variability in daily reactivity. For the multilevel modeling, it is not possible to calculate expected power without making assumptions about the covariance matrix, because work on police surveillance and sleep of this kind is still exploratory. However, within-subjects models tend to be more robust in their assumptions and require a smaller sample, compared with between-subject analyses. For example, assuming a repeated measures analysis of variance with 14 measurements per person, a diary sample size of 72 participants is sufficiently powered (0.85) to detect a small effect size (ƒ=0.1).

## Results

The study received funding in July 2024. Data collection commenced in September 2024. Data collection is underway, and the results are forthcoming. As of August 2025, we have enrolled more than 100 participants in the baseline survey; approximately 35 randomly selected participants have also consented to the diary study.

## Discussion

### Principal Findings

The study is important in that it is one of the few to consider the role of overt and covert policing on sleep health within a single city (where all neighborhoods share the same law enforcement policies), and how these factors vary among neighborhoods based on their socioeconomic and racial or ethnic makeup. Moreover, this study will help elucidate the pathways by which residential disadvantage is associated with sleep health. Furthermore, using a 1-week diary study should help us to better understand the real-time impact of policing and crime on mental health and sleep health across different neighborhood contexts.

### Dissemination Plan

Findings will be disseminated broadly at professional conferences and in peer-reviewed publications. Given that we are at a primarily undergraduate institution (PUI) that is also a commuter campus, this study presents a unique opportunity to train future scholars in the areas of sleep health, health disparities, and health psychology. Thus, some of our dissemination efforts will include our student research assistants and will target student-oriented conferences, presentation opportunities, and peer-reviewed journals (eg, *Psi Chi Journal of Psychological Research*). We also plan to share a summary of the findings with the NYC community boards serving the NTAs where the data are collected.

### Limitations

Funding and time constraints restricted our ability to make the study materials available in other languages. We recognize that the exclusion of non-English–speaking NYC residents, particularly those who speak Spanish, will potentially limit generalizability. Previous research has identified budget constraints, as well as interpretation and translation services, among others, as barriers to the inclusion of participants who speak a language other than English [71]. To mitigate these concerns and capture perspectives of non-English–speaking persons in future studies, it is recommended that researchers plan for interpretation and translation of study materials in grant proposal budgets and seek institutional guidance for accessing these critical services [71]. We further acknowledge that Spanish-speaking or bilingual residents might be concerned about participating due to their immigration status; thus, we do not require anyone to disclose their immigration status.

Although eligible participants must own a smartphone to complete the daily diary, potentially reducing generalizability, recent estimates by the Pew Research Center suggest that 91% of US adults own a smartphone [39]. Additionally, our use of self-report measures for sleep and policing experiences is subject to recall bias. However, this risk is mitigated by using daily diary data. Recruitment efforts that are entirely based on street-intercept can be hindered by external factors, such as poor weather conditions (eg, too cold, too hot and humid, rain, or snow), lack of traffic or slow foot traffic, as well as few or limited public spaces within particular NTAs that are conducive to resident traffic (eg, fewer green spaces). Lack of traffic or slow foot traffic may also be related to the timing of our recruitment shifts. For example, late morning shifts in one NTA may be better for recruitment than late morning shifts in another NTA. Finally, our street-intercept recruitment approach does not document the number of persons approached during a shift. We made the decision not to document this because our eligibility criteria are relatively strict (ie, live in the designated NTA; comfortably fluent in English) and there are numerous instances where “no” responses are not necessarily a lack of study interest, but merely reflect ineligibility prior to official screening. Meaning, our population of interest is never fully represented by those who are available during any given recruitment shift. For example, public parks in NYC are inviting to, and often frequented by, individuals who may not live in the surrounding area (ie, the NTA).

### Conclusions

The study is expected to be useful for identifying populations and residential settings that are most at risk for poor sleep health and should inform future interventions. Despite the potential recruitment limitations, data collection is ongoing, demonstrating the feasibility of this study’s protocol.

## Appendix

Multimedia Appendix 1 Funding proposal reviewer comments.

## Abbreviations

ICE: Index of Concentration at the Extremes
NTA: neighborhood tabulation area
NYC: New York City
PUI: primarily undergraduate institution
SES: socioeconomic status

## Data Availability

The datasets generated or analyzed during this study are not publicly available due to the ongoing nature of the study but may be available from the corresponding author at the conclusion of the study on reasonable request.
