# Supplementary material for: Neighborhood Differences in Omnipresent Policing and Sleep Health in New York City: Protocol for a Multimethod Quantitative Study
Source: JMIR Res Protoc. 2025 Dec 30;14:e82605. doi: 10.2196/82605 (PMC12800734; doi:10.2196/82605)
Supplement: Multimedia Appendix 1 [file resprot_v14i1e82605_app1.pdf]

PSC-CUNY Research Awards (Enhanced): ENHC-55-86

VIEW ABSTRACT CLOSE

	Certified
View Evaluation	✓
View Evaluation	✓

PREVIOUS

PSC-CUNY Research Awards (Enhanced): ENHC-55-86

VIEW ABSTRACT | CLOSE

	Certified
View Evaluation	✓ ✓

HIDE

Evaluation Notes:

Significance and originality of the proposal.
- This work has both scholarly and community-based significance. I am aware of few scholarly studies that have the potential to analyze at this depth the impact that policing has on sleep in communities of color. I also know that the the environmental impact of police surveillance - for example through very bright lights placed in public housing courtyards - is a common complaint I've heard from community-based organizations over the last five years.

Critical analysis of scholarly and/or scientific merit of the proposal.
- I'm very confident that this work has scholarly/scientific merit. The methods are innovative, sophisticated and appropriate. The theoretical model is robust, and this work will further contribute to its robustness. I look forward to reading/using the outcome of this work.

Appropriateness of the requested budget.
- Very appropriate

Constructive recommendations when being critical of the proposal.
-None - this is a very strong study

PREVIOUS

PSC-CUNY Research Awards (Enhanced): ENHC-55-86

VIEW ABSTRACT CLOSE

	Certified
View Evaluation	✓ ✓

HIDE

Evaluation Notes:

This is a terrific and important proposal. While many scholars have documented the structural/economic/educational consequences of racialized policing, few have interrogated in such a fine tuned way the health consequences. This proposal offers creative multi-method design to interrogate how omnipresent policing saturates the body. A new volume by Arline Geronimus called Weathering - how racism penetrates racialized bodies, may be of interest and of course the scholarship conducted by Professor Brett Stoudt at the CUNY graduate center on policing. Terrific work - just the kind of scholarship the PSC CUNY grants should promote.

PREVIOUS
